# The War Efficiency of the Medical Volunteer

**Published:** 1907-12-14

**Authors:** 


### The Royal Army Medical Corps Section.

### THE WAR EFFICIENCY OF THE MEDICAL VOLUNTEER.

We have already in these columns incidentally alluded to the question of practical instruction for the medical units of the Volunteer force, and the great lack of facilities afforded them for carrying out their training on the basis of real war efficiency. While the system of cast-iron regulations, which has fostered the perpetuation of inefficient training, is to be deplored, it is satisfactory to reflect that we have in Sir Alfred Keogh, the Director-General of the Army Medical Service, a keen supporter of the principle of practical instruction, while there are many other willing, able and energetic officers in the Service who are fully alive to the needs of the situation, and who are prepared to carry out these principles on sounder lines than has hitherto been done. The main difficulties in the way of a more satisfactory solution of this vexing problem of efficient training are, of course, financial, but under an improved system these difficulties may be reduced to a minimum, and, as a result, a much superior article obtained for the outlay. The quality of an investment is judged not by any high sounding title, but by the nature of the return afforded on the capital; and while it cannot be said that in the particular case under review the capital is all that could be desired, yet it must be conceded that the comparative return might, without difficulty, be considerably improved. The fault has lain in the system which keeps the main purpose of the department ever overshadowed.

We have alluded previously to particular instances in which commanding officers of units have exercised their initiative, and have attempted to bring the training of their men into line with potential requirements. As a notable example of what might be done towards improving the war efficiency of a medical unit we might detail the training offered to the Glasgow Companies of the Royal Army Medical Corps Volunteers, who have gone further in this direction than any other unit. Colonel Beatson, C.B., the officer commanding, is a strong supporter of the real efficiency of a medical corps, and the lines on which he seeks to train his men show his perception of the real issue at stake. The aim is to fit these men, not to march in line or column or echelon, but to take their places individually in the field ambulance or in a hospital ward in time of stress, and to carry out their duties there for the benefit of the sick or wounded inmates.

With that aim in view the men, after being well grounded in first aid and bearer work, are put through a systematic course of nursing and hygiene. The lectures are delivered by the company officers to each company, and the syllabus is as follows:—

Lecture 1. Introductory remarks as to what sick nursing entails on those undertaking it, especially as to its duties and responsibilities. The management of the sick wards as to warming, ventilation, lighting, and furniture, with special reference to the arrangements of the bed and bedding for both medical and surgical cases.

Lecture 2. Infectious and non-infectious cases. Disinfecting, disinfectants, and antiseptics.

Lecture 3. Details of Nursing:—(1) Administration of medicine, food, and stimulants; (2) Washing and dressing patients; (3) Lifting helpless patients; (4) Prevention of bedsores and their treatment.

Lecture 4. Details of Nursing continued:—(1) Taking temperature, pulse, and respiration, with their record on charts; (2) baths; (3) application of local remedies as poultices, blisters, fomentations, leeches, ointments, etc.; (4) use of sick utensils.

Lecture 5. Observation of the sick as to—skin, sleep, pain, postures, rigors, appetite, vomiting, bowels, cough, and effects of remedies; Management of delirious patients.

Lecture 6. Surgical dressings and their preparation; Assistance at operations; Hints on sick diet; Personal cleanliness; Hygiene.

Those lectures are made additionally interesting, and instructive to the men by lantern illustrations and practical demonstrations, and a large proportion of the members have attended special lectures in one of the out-patient theatres of the Glasgow Western Infirmary, where the most up-to-date apparatus has been demonstrated and shown in actual use.

After this theoretical instruction the men are ready for its practical application under the guidance of experienced orderlies, and this is obtained at Glasgow in the Military Station Hospital at Maryhill and during camp at Netley Hospital. Last summer this very desirable hospital practice at camp was not obtained owing to another cast-iron War Office regulation that Scottish corps must train in Scotland. The rationale of applying such a regulation to a special body of men is difficult to fathom, but applied it was, and as a consequence of this pseudo-economy 250 men encamped at Stobs, and during the week performed work—Bearer Company work—which could be equally well performed on their own drill-field, thus negativing almost all the substantial advantages of the usual Netley camp, when 400 men would have done hospital duty with regulars.

In previous seasons the week at Netley was taken full advantage of. The men encamped behind the hospital and had the experience of tent work, while each morning detachments were detailed for hospital duty, and were sent to the medical and surgical wards to have practical training. During the South African war period these men manned the hospital train, unloaded transports of their sick at Southampton, and did duty in the Doecker hut hospitals, such as they would have done on service.

The course at Netley was arranged as follows:—Distributed to the various wards, the men took part in the regular work of the ward, under the guidance of the regular orderlies, going round with the officer in charge at his morning visit, and in the surgical division seeing the dressing of cases, the preparation of the operating room, and so on.

The routine of the hospital consisted of three parts—

I. Lectures by the Matron.
II. Practical Work in the Wards.
III. Practical Instruction given by the Sisters.

The detail of these various parts is interesting, because it shows how much can, by method and arrangement, be compressed into a very short time.

Lectures—

I. Monday, July 18—Ward Management.
II. Tuesday, July 19—The Care of the Sick.
III. Wednesday, July 20—Principles of Diet.
IV. Thursday, July 21—Germs and Infection.

Practical Work (Daily) :—Making beds; Washing bed-ridden patients; Sweeping and dusting wards; Bringing up breakfasts and serving them.

Practical Instruction (Daily) :—The taking of temperature, pulse, and respiration; The giving of medicine, making poultices, wringing out fomentations and applying them; Padding splints; Dressing surgical cases; Feeding medical cases.

Here is an almost complete epitome of the duties of a ward orderly given in five days.

The instruction at Maryhill Station Hospital during the winter months consists of a week-end tour of duty for selected squads of men who are able to spend the Saturday and Sunday in hospital without undue interference with their regular employment. The squad reports itself for duty on Saturday afternoon. During their period of duty the men obtain quarters in the hospital, and are taken round the wards and shown the store rooms, dispensary, pack store, disinfecting chamber, and other departments, and are instructed by the sister as to measuring medicines, taking temperatures, etc. They serve the breakfasts, assist in the management of the ward, and take the regular routine of orderly work till Sunday evening. They assist with surgical dressings, and on occasion may see an operation, or be present at a post-mortem examination. There is no prescribed course; they simply take what comes. All of this is a decided step towards real war efficiency and is a practical experience of military medical service.

Lastly, there is the important subject of sanitation in field and camp, which is so essential for a medical corps. Special lectures are delivered during Spring, and the application of the instruction is demonstrated at camp. The lectures are four in number, but the increased interest in the subject may result in a further development. The subjects are—

Lecture 1. General consideration of bacteria, and of diseases caused by them.

Lecture 2. Water supply, its importance, and means of obtaining a pure water.

Lecture 3. Sanitation of Camps and disposal of refuse.

Lecture 4. Personal hygiene on the march.

Here there is a corps attempting to qualify itself for service should the necessity arise. It shows what may be done by taking advantage of opportunities. The men, however, are not called upon to undergo such special training to earn the capitation grant: for units which do not so train obtain an equal grant. This is the fault of the system, a system which does not secure the best return on the investment. There is no question of payment by results. Is the old system business? We can but hope that under the new scheme medical units will be encouraged to train as medical units, and that "War efficiency" will be made the test for earning the capitation grant rather than the showy ceremonial drill of the parade-ground.

